# Effect of increased water intake on plasma copeptin in healthy adults

**DOI:** 10.1007/s00394-017-1471-6

**Published:** 2017-06-03

**Authors:** Guillaume Lemetais, Olle Melander, Mariacristina Vecchio, Jeanne H. Bottin, Sofia Enhörning, Erica T. Perrier

**Affiliations:** 1 Hydration and Health Department, Danone Research, Route Départementale 128, 91767 Palaiseau, France; 2 Department of Clinical Sciences, Lund University, Skåne University Hospital, Malmö, Sweden; 3 Department of Internal Medicine, Skåne University Hospital, Malmö, Sweden; 4 Department of Clinical Physiology, Skåne University Hospital, Malmö, Sweden

**Keywords:** Copeptin, Fluid intake, Hydration, Urine osmolality, Water intake

## Abstract

**Purpose:**

Inter-individual variation in median plasma copeptin is associated with incident type 2 diabetes mellitus, progression of chronic kidney disease, and cardiovascular events. In this study, we examined whether 24-h urine osmolality was associated with plasma copeptin and whether increasing daily water intake could impact circulating plasma copeptin.

**Methods:**

This trial was a prospective study conducted at a single investigating center. Eighty-two healthy adults (age 23.6 ± 2.9 years, BMI 22.2 ± 1.5 kg/m^2^, 50% female) were stratified based upon habitual daily fluid intake volumes: arm A (50–80% of EFSA dietary reference values), arm B (81–120%), and arm C (121–200%). Following a baseline visit, arms A and B increased their water intake to match arm C for a period of 6 consecutive weeks.

**Results:**

At baseline, plasma copeptin was positively and significantly associated with 24-h urine osmolality (*p* = 0.002) and 24-h urine specific gravity (*p* = 0.003) but not with plasma osmolality (*p* = 0.18), 24-h urine creatinine (*p* = 0.09), and total fluid intake (*p* = 0.52). Over the 6-week follow-up, copeptin decreased significantly from 5.18 (3.3;7.4) to 3.90 (2.7;5.7) pmol/L (*p* = 0.012), while urine osmolality and urine specific gravity decreased from 591 ± 206 to 364 ± 117 mOsm/kg (*p* < 0.001) and from 1.016 ± 0.005 to 1.010 ± 0.004 (*p* < 0.001), respectively.

**Conclusions:**

At baseline, circulating levels of copeptin were positively associated with 24-h urine concentration in healthy young subjects with various fluid intakes. Moreover, this study shows, for the first time, that increased water intake over 6 weeks results in an attenuation of circulating copeptin.

**Clinical Trial Registration Number:**

NCT02044679.

## Introduction

Vasopressin (AVP) is a peptide hormone produced in the hypothalamus that influences body water balance by exerting an anti-diuretic effect. Copeptin, the C-terminal segment of the prohormone pre-pro-vasopressin, has recently gained popularity as a surrogate marker of circulating AVP concentration. This is due in part to the fact that the half-life of plasma AVP is very short; copeptin, which is more stable, may, in fact, better represent plasma AVP secretion, since it is produced in a 1:1 ratio with AVP. Previous studies have demonstrated that plasma copeptin (*P*_Cop_) is positively and independently associated with incident type 2 diabetes mellitus [1–3], metabolic syndrome [4, 5], progression of chronic kidney disease [6–9], and cardiovascular disease [10–12].

The osmotic stimulation of AVP and copeptin secretion is well documented, and the normal physiological response is for AVP to increase in response to plasma hyperosmolality, decreased arterial pressure, or decreased blood volume [13]. AVP acts via V2 receptors in the nephron to increase water reabsorption, trigger antidiuresis, and maintain total body water homeostasis. In the literature, the osmotic threshold for secretion has been reported as varying from 280 to 295 mOsm/kg [13–15]. However, although the regulation of AVP or copeptin secretion has been extensively studied in situations where water balance is substantially challenged, such as progressive dehydration, or hemorrhage, less is known about the relationship between copeptin, urinary hydration biomarkers, and fluid intake in the general population under normal daily living conditions, where variation in water balance is more likely due to differences in fluid intake. In the general population, measures of urine concentration have been described as well suited to detect differences in the daily hydration process [16]. Among these, 24-h urine osmolality (*U*_Osm_) and 24-h urine specific gravity (*U*_SG_) are positively associated with increased water retention by the kidney [17]. In the literature, AVP was positively and significantly correlated with spot urine osmolality in healthy recumbent subjects [18]. A recent paper also described a higher concentration of AVP in people maintaining a 24-h *U*_Osm_ above 500 mOsm/kg [19]. Similarly, a significant direct correlation was described between *P*_Cop_ and calculated [8] or measured [20] osmolality of spot urine, in healthy people. However, to the best of our knowledge, it has never been demonstrated that modifying daily fluid intake can change *P*_Cop_ concentration in young, healthy adults with no evidence of metabolic or renal disorders. Given the reported associations between elevated *P*_Cop_ and cardiometabolic and kidney disease, increased water intake may play a preventive role. As a first attempt to characterize this effect, we sought to investigate the association between copeptin and 24-h *U*_Osm_, as well as to determine whether simply increasing daily water intake might reduce circulating *P*_Cop_.

## Materials and methods

This prospective study was conducted at a single investigating center (Biotrial, Rennes, France) according to the ethical principles stated in the revised version of the Declaration of Helsinki [21]. This is a secondary analysis of data from a study whose primary purpose was to investigate the equivalence between spot and 24-h urinary hydration biomarkers [22]. All subjects provided written informed consent and the study was approved by the local Ethics Committee of Nancy, France (Comité de Protection des Personnes Est-III, Nancy, France). The study was registered on clinicaltrials.gov (NCT02044679). Subjects were recruited between October 2013 and February 2014, and study data were collected between October 2013 and March 2014. This study was carried out in free-living subjects who agreed to refrain from intensive physical activity during the study period (from the screening visit until the end of study). The exclusion criteria included following a vegetarian diet, smoking >10 cigarettes/day, excessive alcohol consumption (>20 g alcohol/day), pregnancy or breastfeeding, use of any prescription or OTC medication which may have interfered with water balance or metabolism within 14 days before the start of the study and any clinically relevant acute or chronic diseases.

Recruitment was stratified to obtain a sample with a range of habitual fluid intake. Participants completed a 3-day electronic food and fluid diary (NutriSaas-24WQ-waters; MXS, France) and were subsequently allocated to three arms based upon habitual daily fluid intake volume: arm A, subjects with a fluid intake ranging from 50 to 80% of EFSA dietary reference values for water from fluids (i.e., 2.0 L/day for females and 2.5 L/day for males) [23]; arm B, fluid intake between 80 and 120% of EFSA dietary reference values; and arm C, fluid intake between 120 and 200% of EFSA dietary reference values. To ensure that included participants were consistent in their daily drinking habits, any participant whose daily fluid intake volume varied by more than 25% across the three collection days was subsequently excluded from the study.

All participants completed a baseline visit. Next, arms A and B completed a 6-week water intake intervention designed to increase their intake to match the baseline fluid intake volume of arm C. Participants in arm B were provided with 1 L/day (women) or 1.5 L/day (men) of Evian^®^ natural mineral water, while those in arm A, whose baseline intake was the lowest, were provided with 1.5 L/day (women) or 2 L/day (men) in addition to their usual fluid intake. Participants were advised not to change their habitual fluid intake except for the water provided as part of the intervention. Compliance was encouraged and monitored through a daily paper log in which participants accounted for each bottle provided, and noted whether each bottle was fully consumed, partially consumed, or not consumed at all. These paper logs were returned to the study site every 2 weeks. Furthermore, text messaging and telephone calls were used to remind patients to follow the intervention and complete the diaries.

At baseline (arms A, B, and C), and during three biweekly follow-up visits (+2, +4, and +6 weeks; arms A and B only), participants completed an electronic food and fluid diary, during the 3 days preceding the laboratory visit, collected 24-h urine samples at home the day before each laboratory visit, and reported to the lab for a fasted morning blood draw. Urine and plasma osmolality were measured using a freezing point depression osmometer (Advanced Model 2020 Multi-Sample Osmometer; Advanced Instruments, Inc., Norwood, MA, USA). *U*_SG_ was measured with a refractometer (Pen Urine S.G., Atago Ltd.). *P*_Cop_ concentration was measured in a single batch with a sandwich immunoassay (B.R.A.H.A.M.S. Copeptin US Kryptor; Thermo Scientific, Germany). Urine creatinine concentration (*U*_Creat_) was measured using a kinetic modification of the Jaffe procedure using a Unicel DxI 600 (Beckman Coulter Inc., Fullerton, CA, USA).

The data are expressed as mean ± SD for continuous variables or median with the 25th and 75th percentiles for nonparametric values (*P*_Cop_). To evaluate baseline relationships between *P*_Cop_ and total fluid intake (TFI), *P*_Osm_, 24-h *U*_Osm_, *U*_SG_, or *U*_Creat_, two linear regression mixed models were calculated: (1) non-adjusted (Model 1); (2) adjusted for age, sex, smoking, and BMI (Model 2). To evaluate the effect of the 6-week water intervention on *P*_Cop_ and urine osmolality, a one-way ANOVA and Kruskal–Wallis test were performed. The level of statistical significance was fixed at *p* < 0.05. Statistical analysis was performed using SAS (v.9.1.3; Cary, NC, USA).

## Results

**Fig. 1:**
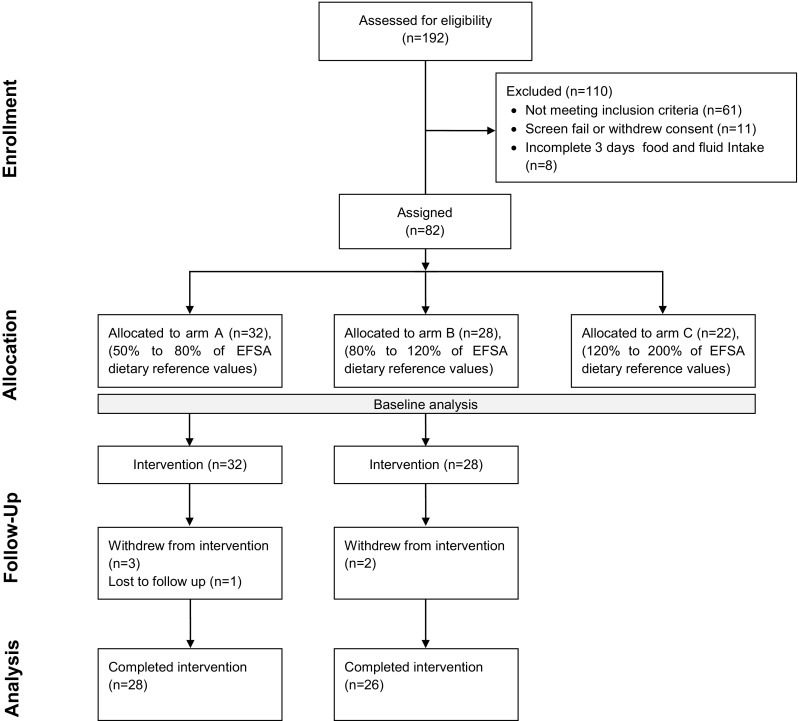
Study flow diagram

The final sample included 82 participants (arm A, *n* = 32; arm B, *n* = 28; arm C, *n* = 22; age 23.6 ± 2.9 years; BMI 22.2 ± 1.5 kg/m^2^; 50% female; see Fig. 1). TFI and plain water intake at baseline were significantly different between arms, as was intended by the stratified recruitment.

Neither baseline BMI, energy intake, nor consumption of other beverage categories (for instance, sugar-sweetened beverages (SSB) or caffeinated beverages) differed between groups (Table 1). At +2, +4, and +6 weeks of the water intake intervention in arms A and B, reported TFI increased to 2786 ± 618, 2760 ± 559, and 2723 ± 594 mL/day, suggesting that the intervention was successful in increasing TFI to meet or exceed the baseline intake of arm C. Moreover, with a reported compliance of 94 ± 6% for consumption of the water provided, as documented in the daily log described previously, the consumption of plain water as a proportion of TFI was also similar between arm C at baseline, and arms A and B during the intervention. As expected, no changes in the consumption of other beverages were observed during the intervention in both groups.

**Table 1:** Baseline characteristics of the study participants (ITT population)

	Total (*n* = 82)	Stratification arm		
		A (*n* = 32)	B (*n* = 28)	C (*n* = 22)
Age (years)	23.6 ± 3.1	23.6 ± 3.1	23.3 ± 2.8	23.9 ± 2.8
Gender (male/female)	41/41	20/12	13/15	8/14
BMI (kg/m^2^)	22.2 ± 1.5	22.0 ± 1.6	22.2 ± 1.6	22.4 ± 1.4
Energy intake (kcal/day)	1781 ± 554	1715 ± 588	1787 ± 534	1868 ± 539
Total fluid intake (mL/day)*	1830 ± 648	1430 ± 490	1826 ± 405	2418 ± 671
Drinking water (mL/day)*	1012 ± 582	788 ± 417	975 ± 638	1386 ± 552
Sugar sweetened beverages (mL/day)	299 ± 332	277 ± 273	294 ± 416	335 ± 301
Plasma copeptin (pmol/L)	5.10 [3.3;6.8]	5.28 [3.3;7.5]	5.23 [3.5;7.6]	4.20 [3.2;5.9]
Plasma osmolality (mOsm/kg)	293 ± 4.5	293 ± 4.1	293 ± 5.0	292 ± 4.3
24-h urine osmolality (mOsm/kg)*	567 ± 213	634 ± 211	554 ± 203	486 ± 207
24-h USG*	1.015 ± 0.006	1.017 ± 0.005	1.015 ± 0.006	1.013 ± 0.005
24-h urine creatinine (mmol)	12.8 ± 3.8	13.2 ± 3.4	12.4 ± 4.4	12.9 ± 3.7

Continuous variables are expressed as the mean ± SD or median and IQR (25th–75th percentiles) as appropriate

*BMI* body mass index; *ITT* intent-to-treat

* Statistically significant differences between arms (*p* < 0.05; ANOVA)

**Fig. 2:**
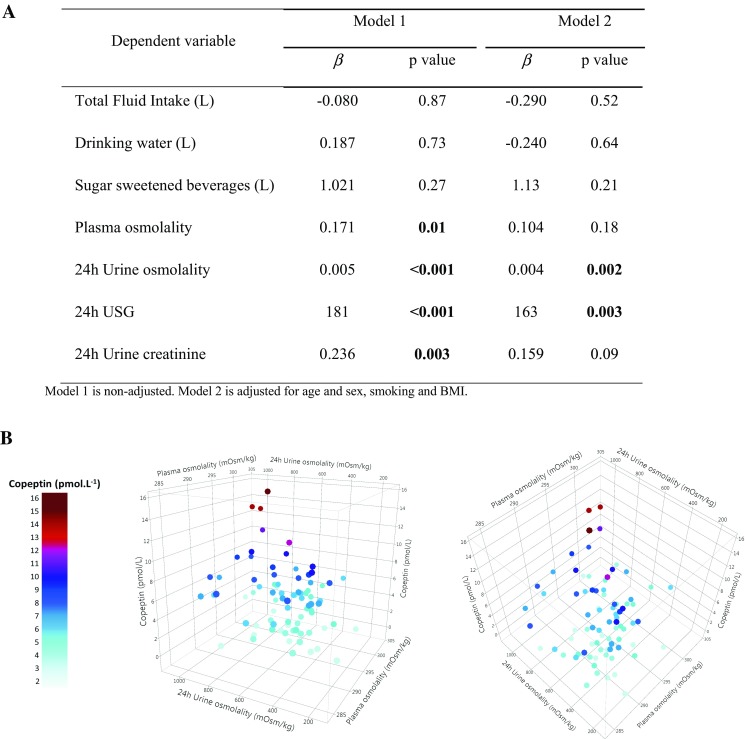
Association with copeptin at baseline. **a** Multivariable regression analysis with copeptin as the dependent variable. **b** 3D plot displaying the correlation between copeptin, *P*_Osm_, and 24-h *U*_Osm_ presented as viewed from the front (left) and from above (*right*). Circulating copeptin increases with increasing 24-h *U*_Osm_ and *P*_Osm_. The *light-blue* to *dark-red color* gradient reflects the lower to higher copeptin concentration

Linear regression analysis was performed to model the baseline *P*_Cop_ as a function of hydration variables. Figure 2a shows the *β* coefficients in two multivariate models. At baseline, higher *P*_Cop_ was associated with higher *P*_Osm_, higher 24-h *U*_Osm_, higher 24-h *U*_SG_, and higher 24-h *U*_Creat_ in the non-adjusted model. *P*_Osm_ and 24-h *U*_Creat_ did not remain associated with copeptin after adjustment. On the contrary, 24-h *U*_Osm_ and 24-h *U*_SG_ were positively associated with *P*_Cop_ in both models. Concretely, on average, *P*_Cop_ increased significantly with increased 24-h *U*_Osm_ (0.43 pmol/L for each 100 mOsm/kg change; *p* = 0.002) and 24-h *U*_SG_ (0.82 pmol/L for each 0.005 change; *p* = 0.003), in the fully adjusted model. TFI, drinking water, and SSB were not associated with copeptin. Figure 2b presents a 3D plot of copeptin vs 24-h *U*_Osm_ and *P*_Osm_ at baseline.

Table 2 presents *P*_Cop_ and hydration variables at baseline and follow-up among subjects who received the study product. During the 6-week follow-up, 24-h *U*_Osm_ decreased for arms A and B. For both groups, this reduction remained stable during the follow-up period, from 591 ± 206 mOsm/kg at baseline to 339 ± 109, 351 ± 156, and 364 ± 117 mOsm/kg at +2 weeks (*p* < 0.001), +4 weeks (*p* < 0.001), and +6 weeks (*p* < 0.001), respectively. Similarly, *U*_SG_ decreased significantly from 1.016 ± 0.005 to 1.010 ± 0.004 (*p* < 0.001). Copeptin decreased from 5.18 to 4.45, 4.10, and 3.90 pmol/L, after +2 weeks (*p* = 0.019), +4 weeks (*p* = 0.047), and +6 weeks (*p* = 0.012), respectively. Copeptin concentration decreased between baseline and week 6, reaching a 24.7% decrease after 6 weeks.

**Table 2:** Effect of increased water intake on 24-h U_SG_, 24-h *U*_Osm_, and copeptin (ITT population)

	Baseline^ab^	+2 weeks	+4 weeks	+6 weeks
Total fluid intake, mL/day (SD)				
Arm A	1444 (491)	2772 (526)	2823 (580)	2747 (510)
Arm B	1826 (405)	2802 (716)	2690 (538)	2698 (682)
All	1625 (488)	2786* (618)	2760* (559)	2723* (594)
Drinking water, mL/day (SD)				
Arm A	790 (423)	2158 (601)	2259 (734)	2226 (537)
Arm B	975 (638)	1966 (822)	1957 (694)	1959 (857)
All	878 (539)	2067* (714)	2116* (725)	2097* (715)
Mean 24-h USG (SD)				
Arm A	1.017 (0.005)	1.009 (0.003)	1.010 (0.004)	1.009 (0.003)
Arm B	1.015 (0.006)	1.010 (0.003)	1.011 (0.004)	1.011 (0.004)
All	1.016 (0.005)	1.010* (0.003)	1.010* (0.004)	1.010* (0.004)
Mean 24-h *U*_Osm_, mOsm/kg (SD)				
Arm A	624 (206)	321 (110)	329 (165)	343 (100)
Arm B	554 (203)	360 (105)	376 (144)	387 (131)
All	591 (206)	339* (109)	351* (156)	364* (117)
Median copeptin, pmol/L (IQR)				
Arm A	5.15 (3.3;7.4)	4.35 (3.2;5.9)	4.25 (3.0;6.6)	3.93 (3.0;5.6)
Arm B	5.20 (3.5;7.5)	4.63 (2.9;5.8)	4.05 (3.4;6.0)	3.90 (2.7;5.7)
All	5.18 (3.3;7.4)	4.45* (3.0;5.9)	4.10* (3.2;6.3)	3.90* (2.7;5.7)

*IQR* inter-quartile range, *SD* standard deviation, *ITT* intent-to-treat

^a^In Arm A, only 31 on 32 subjects received the study product

^b^In Arm B, one missing value of copeptin

* Statistically significant compared to baseline (*p* < 0.05; ANOVA or Kruskal–Wallis test)

## Discussion

In this trial, we found that *P*_Cop_, a surrogate marker of vasopressin, is associated with 24-h *U*_Osm_ in healthy subjects. Moreover, in individuals habitually consuming low-to-moderate TFI, a 6-week intervention of increased plain water intake significantly reduced circulating median copeptin. This finding suggests, for the first time, that increased water intake may be a very simple and inexpensive intervention for the reduction of circulating vasopressin. Although these results need to be further confirmed by randomized controlled trials, they may be of public health interest, as several prospective studies have identified elevated plasma concentration of copeptin as an independent risk factor for development of new-onset diabetes mellitus, decline of renal function, and cardiovascular disease [2, 3, 9–11].

The present study demonstrates an association between urine concentration, measured by 24-h *U*_Osm_ or 24-h *U*_SG_, and *P*_Cop_. This finding complements the results of previous studies showing associations between copeptin and spot morning urine osmolality [8, 20], and suggests that individuals with concentrated 24-h urine may have persistently higher circulating vasopressin. Recently, a population-based study (*n* = 1010) showed that copeptin and 24-h *U*_Osm_ were significantly associated in men (*r* ^2^ = 0.44; *p* < 0,001) and women (*r* ^2^ = 0.37; *p* < 0,001) [24]. Quantitatively, an incremental increase of 1 pmol/L in copeptin concentration is associated with ~200 mOsm/kg higher 24-h *U*_Osm_ and a *U*_SG_ increase of 0.006. Figure 2b shows the relationship between *P*_Osm_, 24-h *U*_Osm_, and copeptin. While *P*_Osm_ remains the major determinant of osmotic copeptin release, significant copeptin variations can also be observed with variation in urinary concentration, highlighting the renal response to small neuroendocrine variations. It is of interest to note the lack of association between plasma copeptin and *P*_Osm_ (Fig. 2a). Our results suggest that in the general population in normal daily conditions—i.e., in the absence of uncompensated water loss—24-h *U*_Osm_ may best reflect circulating copeptin. Moreover, the association between copeptin and 24-h *U*_Osm_ remains significant after adjustment for *P*_Osm_ (data not shown). The absence of difference in *P*_Osm_ between high and low drinkers, with a significantly different circulating AVP, has already been described elsewhere [25] and indicates a sensitive regulation of urine concentration, through AVP secretion, to preserve *P*_Osm_. Thus, the relatively broad range of urine osmolality observed in healthy subjects may facilitate the study of the physiological impact of small changes in copeptin, by providing an easy screening tool for identifying individuals who are more likely to have higher circulating copeptin. Moreover, increased water intake during a relatively short intervention period (6 weeks) decreased circulating copeptin by 24.7%. Copeptin values measured after intervention (3.93 and 3.90 pmol/L, in arms A and B, respectively) are even lower than those measured at baseline in arm C (4.20 pmol/L). These findings may be due to a higher TFI consumption in arm A and arm B during the intervention (2.8 and 2.7 L/day vs 2.4 L/day in arm C) and a higher contribution of drinking water to TFI. Increasing water intake may thus represent a simple way to reduce circulating copeptin under normal daily living conditions. At baseline, TFI was not significantly associated with circulating copeptin (Fig. 2a). This may be explained, partly, by the opposite effects of each fluid on vasopressin: a negative association with drinking water and a positive association with SSB. The small sample size of this study did not allow to reach statistical significance, but this tendency has already been observed in the literature [8].

In several recent cohort studies, higher circulating copeptin has been associated with the development of metabolic disorders. In an analysis conducted on a subset of participants in the Malmö diet and cancer study, higher baseline plasma copeptin was associated with increased prevalence of type 2 diabetes mellitus [2]. More convincingly, health status at the 12-year follow-up was suggestive of a directional link between copeptin and onset of metabolic disorders: among subjects without type 2 diabetes mellitus or impaired fasting glucose at baseline, those who became diabetic during the follow-up period had higher baseline plasma copeptin (6.74 vs 4.90 pmol/L) than nonconverters. Simply put, elevated copeptin preceded the diagnosis of the disease; moreover, adding copeptin to classic diabetes risk factors improved the discriminative ability of both personal and clinical models of diabetes prediction. In another human trial, median copeptin higher than 5 pmol/L has been significantly associated with increased risk of developing the metabolic syndrome and insulin resistance [5]. These findings suggest that while the changes in plasma copeptin with increased water intake are relatively small in the present study (i.e., from 5.18 to 3.90 pmol/L), it is entirely plausible that this small reduction is of clinical relevance. This also suggests that individuals with chronically elevated 24-h urine osmolality, representing sustained anti-diuretic activity, may be at higher risk for metabolic disease. Two recent papers suggest U500 (24-h *U*_Osm_ ≤ 500) and U800 (24-h *U*_Osm_ ≥ 800) as thresholds for optimal hydration and mild dehydration, respectively [19, 26]. Applying these thresholds to the data visually represented in Fig. 2, median copeptin at baseline was 4.30 pmol/L (*n* = 36) for individuals below the U500 cutoff, and 6.93 pmol/L (*n* = 12) for individuals above U800. This latter value is in line with thresholds associated with increased risk of incident diabetes in the literature, 6.74 and 6.79 pmol/L, respectively [2, 3].

Previous studies have already demonstrated a beneficial effect of increased water intake on reducing the risk of renal pathologies, such as kidney stones or chronic kidney disease, and cardiovascular disease [27–29]. From the existing literature and our clinical findings, it seems that increasing plain water intake has the potential to play a role in reducing metabolic disease risk. The presence of vasopressin receptors V1a and V1b in the liver and pancreas, respectively, adds a plausible mechanistic explanation to a link between water intake, copeptin, and metabolic disease, which needs to be further investigated. Moreover, as copeptin measurement requires substantial expertise and cost, measurement of 24-h urine concentration may represent an alternative to facilitate the early identification of at-risk individuals, and may also serve as a means to track increases in water intake.

In the present study, participants drinking less than 50% or more than 200% of EFSA dietary reference values were excluded by design, which limits the generalization of the findings to the extent to which other populations follow similar drinking patterns. Nevertheless, very low and very high drinkers represent a relatively limited part of the general population. A recent paper evaluating TFI in 13 countries worldwide showed that more than 75% of the general population has a fluid consumption between the lower and upper bounds used in this study [30]. Another methodological limitation is the lack of a control group which limits the extrapolation of the results. However, a study with a similar intervention carried out in patients with chronic kidney disease observed no significant differences in plasma copeptin between baseline and 6 weeks for the control group, suggesting that plasma copeptin would remain relatively stable over a 6-week period in a control group [31].

In summary, the present study provides preliminary evidence that copeptin can be modulated by increasing plain water intake in young, healthy subjects drinking from 50 to 120% of EFSA dietary reference values. Our findings warrant a need for further research, including randomized controlled trials, to evaluate whether water-induced reduction of vasopressin is able to reduce the risk of developing diseases that appear to be associated with higher plasma concentration of copeptin, such as diabetes mellitus, chronic kidney disease, and cardiovascular disease.
